# Supplementary material for: A Modelling Framework Linking Resource-Based Stochastic Translation to the Optimal Design of Synthetic Constructs
Source: Biology (Basel). 2021 Jan 7;10(1):37. doi: 10.3390/biology10010037 (PMC7826857; doi:10.3390/biology10010037)
Supplement: Supplementary file 1 [file biology-10-00037-s001.pdf]

Supplementary Information

In this document we detail all reactions and parameter values used in our stochastic simulations. Following this, we provide additional numerical simulation results obtained by varying parameters related to 'slow codons': the length of mRNA transcripts, and the position and efficiency of slow codons.

S1 Model equations

The reactions used in our model are a stochastic version of those used in [1]. One of the key differences is our implementation of translation, where we use a multi-step reaction scheme instead of a single-step one. In Table S1, we display both the reactions and rate calculations where appropriate. Symbols for variables and parameters are detailed in Tables S2 and S3, respectively. In what follows we define the following elements and their corresponding sets: $x \in \{r, e, q, h\}$, $X \in \{R, E, Q, H\}$, $A \in \{\text{all species except mRNA}\}$.

Table S1. Biochemical reactions and their associated reaction rates.

Process	Reaction	Rate
Nutrient transport	$\emptyset \xrightarrow{k_s(E)} s$	$k_s(E) = \varepsilon_t\, E$
Nutrient metabolism	$s \xrightarrow{k_g(E,s)} g\ n$	$k_g(E,s) = \varepsilon_m\, E\, \dfrac{s^{h_m}}{K_m^{h_m} + s^{h_m}}$
Transcription	$\emptyset \xrightarrow{k_{\mathrm{TX}_x}(g)} x$	$k_{\mathrm{TX}_y}(g) = \dfrac{v_{\mathrm{TX}_y}\, g}{K_{TX_y} + g}$ $k_{\mathrm{TX}_q}(g) = \dfrac{v_{\mathrm{TX}_q}\, g}{K_{TX_q} + g}\, \dfrac{K_I^{h_I}}{K_I^{h_I} + q^{h_I}}$
Translation initiation	$R + x_{-1} \xrightarrow{c_X} x_1$	c_X
Translation elongation	$x_i + g \xrightarrow{k_{\mathrm{TL}_X}(g)} x_{-i,i+1}$	$k_{\mathrm{TL}_X}(g) = \dfrac{v_{\mathrm{TL}}\, g^{h_{\mathrm{TL}}}}{K_{\mathrm{TL}_X}^{h_{\mathrm{TL}}} + g^{h_{\mathrm{TL}}}}\, v_{x_i}$
Translation termination	$x_n + g \xrightarrow{k_{\mathrm{TL}_X}(g)} x_{-n} + R + X$	
Dilution and degradation	$A \xrightarrow{\lambda} \emptyset$ $x \xrightarrow{\lambda + d} \emptyset$	$\lambda = \dfrac{\Delta m}{10^8\, \Delta t}$

S1.1 Formulating growth rate to keep cell mass constant

In this section we detail how growth rate is calculated. We begin with a cell in mid-exponential growth. Over the course of our stochastic simulation steps, new cell mass is continually produced due to protein production. To keep cell mass constant between successive stochastic steps, we find a dilution rate, λ, that balances the increase in cell mass due to protein production. We determine such dilatation rate λ as explained hereafter.

We define M as the cell mass expressed in terms of average amount of amino acids in the cytoplasm. We use indices to denote what the cell mass is at different stages during a stochastic simulation step. Given this, $M_{current}$ denotes the cell's mass at the beginning of a simulation step. M_{bd} denotes cell mass following completion of a reaction during the considered stochastic simulation step (which could involve production of one protein molecule). Finally, M_{new} denotes the mass of the cell following any potential dilution. The change in cell mass during a stochastic simulation step is defined as $\Delta M = (M_{bd} - M_{current})$. To ensure that the cell mass is maintained constant from one stochastic simulation step to the next, we need to impose $M_{new} = M_{current}$. This is achieved by defining $M_{new} = M_{bd} \frac{M_{current}}{M_{current}+\Delta M}$:

$$M_{new} = M_{bd} \frac{M_{current}}{M_{current} + \Delta M} = (M_{current} + \Delta M) \frac{M_{current}}{M_{current} + \Delta M} = M_{current}. \quad \text{(S1)}$$

Defining M_{new} this way is equivalent to considering a specific dilution rate intended to keep cell mass constant between stochastic simulation steps. This dilution rate is defined as $-\frac{\Delta M}{\Delta t}$, i.e. the negative rate of change of cell mass between stochastic steps, under the constraint $M_{new} = M_{current}$:

$$\lambda_{aa} = -\frac{M_{new} - M_{bd}}{\Delta t} = \frac{\Delta M}{\Delta t}. \quad \text{(S2)}$$

In our simulations, we have chosen the initial protein values (see Section S2) so that the total amount of amino acids corresponds to the approximate average number of amino acids in an *E. coli* cell [2], i.e. 10^8 amino acids. Therefore, to express the dilution rate in terms of the mass of one cell, we divide λ_{aa} by this target average number of amino acids per cell:

$$\lambda = \frac{\lambda_{aa}}{10^8} = \frac{\Delta M}{\Delta t \; 10^8}. \quad \text{(S3)}$$

S2 Variables and initial values

The variables used in our model are shown below, alongside the initial values we used in our simulations. Endogenous initial values were chosen by running preliminary deterministic simulations and using their steady-state output as the initial value for the stochastic simulations. Initial values for the heterologous simulations are taken from the steady-state values of an endogenous simulation with a nutrient quality of 100 (no unit).

Table S2. The variables used in the simulations and their initial values.

Variable	Description	Initial value, endogenous (molecules/cell)	Initial value, herologous (molecules/cell)
s	Internal nutrients	128	49
g	Energy unit	100,000	7,699
r	mRNA for R fraction	187	85
e	mRNA for E fraction	278	119
q	mRNA for Q fraction	3,508	2,667
h	mRNA for H fraction	–	0
R	Ribosomal proteins	7,373	5,713
E	Enzyme proteins	10,924	7,995
Q	Housekeeping proteins	138,080	182,505
H	Heterologous proteins	–	0

S3 Parameter values

A list of the parameters used in our simulations is presented in Table 3. Values taken from literature are indicated in the column titled "Source" with the corresponding bibliographic reference(s) indicated in square brackets. Most parameter values are taken from [1]. When values are modified from those proposed in the literature, this is indicated with one or multiple † symbols and the corresponding explanation for this change is presented in Section S3. The following abbreviations are used in Table 3: aa: amino acid. R_f: ribosomal footprint = 30 nucleotides = 10 aa; the span of one ribosome on an mRNA transcript. *Input*: different values within the range shown have been used to conduct stochastic simulations. '–' denotes no unit.

Table S3. Parameter values used in our model.

Parameter	Description	Value	Unit	Source
M_{cell}	Cell mass	10^8	aa	[2] (also in [1])
N_r	Number of ribosomal footprints per r transcript	750	R_f	[3] (also in [1])
$N_e = N_q = N_h$	Number of ribosomal footprints per e, q, h transcript	30	R_f	[4] (also in [1])
n_{aa}	Number of amino acids per ribosomal footprint	10	aa	[5]
$C_R = C_E = C_Q$	Translation initiation rate for R, E, Q fractions	1	–	[6]
v_{TL}	Maximum translation elongation rate	1,260	aa min^{-1}	[2] (also in [1])
h_I	Q fraction autoregulation Hill coefficient	4	–	[6]
d	mRNA degradation rate	0.1 if free, 0 if not	molecs min^{-1}	[6] (also in [1])
v_{TX_h}	Maximum transcription rate for h	$850 \times \mathrm{prom}_h$	molecs min^{-1} cell^{-1}	Chosen relative to prom_h
ε_t	Nutrient transport coefficient	0.363	min^{-1}	†
ε_m	Nutrient metabolism coefficient	2.9	min^{-1}	†
K_m	Nutrient metabolism Hill threshold	100	molecs cell^{-1}	††

K_I	Q fraction autoregulation Hill threshold	5,000	–	††
K_{TX_r}	Transcription Hill threshold for r	30,000	molecs cell^{-1}	††
K_{TX_e}	Transcription Hill threshold for e	3,000	molecs cell^{-1}	††
K_{TL}	Translation elongation Hill threshold	1,000	molecs cell^{-1}	††
K_{TX_h}	Transcription Hill threshold for h	30,000	molecs cell^{-1}	††
K_{TX_q}	Transcription Hill threshold for q	10,000	molecs cell^{-1}	††
h_m	Nutrient metabolism Hill coefficient	3	–	†††
h_{TL}	Transaltion elongation Hill coefficient	2	–	†††
v_{TX_r}	Maximum transcription rate for r	27	molecs min^{-1} cell^{-1}	††††
v_{TX_e}	Maximum transcription rate for e	20	molecs min^{-1} cell^{-1}	††††
n	Nutrient quality	10-600	–	*Input*
prom_h	Promoter strength of gene construct	0.3-3	–	*Input*
RBS_h	RBS strength of gene construct	0.3-3	–	*Input*
v_{x_i}	Relative codon efficiency on transcript x at position i	0.005-1	–	*Input*
C_H	Translation initiation rate for H fraction	RBS_h	molecs min^{-1} cell^{-1}	*Input*

S3.1 Explanation for changes in some values compared to those proposed in [6]

The change of some of the values relative to those proposed [1] is founded on intuitive reasoning explained here-in. We note that the values in [1] also include some uncertainty, and so a detailed parameter fit against an extensive experimental data set would be required to further validate the proposed values. This is however beyond the scope of our model.

Nutrient-related parameters (†)

In [1], parameters ε_t and ε_m take values of 726 min^{-1} and 5,800 min^{-1} respectively, which we reduce by 2000-fold to values of 0.363 min^{-1} and 2.9 min^{-1}. This is for two reasons which are multiplicative: firstly, we vary nutrient quality from 10 to 600 in order to reach saturation of growth rate values in our model output (Figure 4A, main text), which is two to three orders of magnitude higher than in [1]. This would significantly change the dynamics of nutrient transport and metabolism, therefore to maintain the rates of these processes, we first reduce ε_t 1000-fold. Secondly, [1] uses two classes to describe 'enzymatic proteins' with identical transcription and translation dynamics. We combine these into one class, E, meaning that any calculation involving E proteins would be over-represented 2-fold compared to [1]. This requires requires balancing ε_t and ε_m by a further factor of two, yielding a reduction of $1{,}000 \times 2 = 2{,}000$ in total.

Adjusting half-saturation constants (††)

Half-saturation constants (HSCs) are used in Hill equations for reactions involving transcription, translation and nutrient metabolism. In [1], a single parameter is used for each of translation and metabolism, and two are used for transcription due to a separation of ribosomal and non-ribosomal reactions; in our model, we furthermore use transcription HSCs for each protein fraction. Those parameters used for translation and transcription relate to quantities of g ('energy units'), where one unit is subsequently used in each translation elongation step. The HSC values used in [1] for translation

and R/non-R transcription are 7.0, 4.4 and 430 energy units per cell respectively; we deem these as too low for our model, as explained below.

In order to utilise the saturating effects from the Hill equations, the HSCs for transcription and translation should be within a similar order of magnitude as the steady-state value of energy units in the cell. While literature is sparse on average quantities of energy units, we can calculate a value from simple approximations. In our simulations, the number of mRNA molecules for each species at steady state is on the order of ~10^3, and each such molecule requires 30 (for E,Q,H) or 750 (for R) energy units to fully translate, or approximately ~10^2 (see Table 2). We reason that a typical *E. coli* cell in exponential growth would be adapted to store energy reserves (in the form of ATP) to be able to sustain some translational capacity for short periods without any nutrient intake [7]. For example, in order to translate just 1% of its proteome using existing energy reserves only would require approximately $\frac{10^3 \times 10^2}{100} = 10^3$ energy units per cell. This suggests that, in order to be in the energy range where saturation effects from Hill kinetics are applied, the HSCs for transcription and translation should be at least 10^3 in our model. Given this, we increased the values of HSCs for transcription and translation to be in the range 1,000 to 30,000 energy units. Although the precise values are arbirtary, we chose our values to ensure that (i) the HSC for ribosomal transcription was higher than for the other native fractions (i.e. it gets inhibited more readily), and (ii) the lowest value was the HSC for translation (i.e. it gets inhibited less readily). This is to reflect the fact that, in response to low energy levels, an *E. coli* cell inhibits ribosomal RNA biogenesis more significantly than other processes [8].

Adjusting Hill coefficients (†††)

In [1], the Hill coefficients used for nutrient metabolism and translation elongation were both 1, producing a classic Michaelis-Menten saturation curve. We instead wanted these processes to behave more switch-like to reflect the fact that many cellular processes are tightly controlled. To do this, we increased the Hill coefficients from 1 to 3 for nutrient metabolism (h_m) and from 1 to 2 for translation elongation (h_{TL}), meaning that change in kinetics will now occur over a smaller range of values.

Adjusting parameters to reflect assumptions about *E. coli* transcript composition (††††)

In order to make the ratio of e-transcripts to q-transcripts in the cell more realistic, we changed values of v_{TX_e} and v_{TX_q} from 4.14 and 948.93 in [1] to 20 and 850 respectively (unit: molecs min^{-1} cell^{-1}). In both our model and in [1], e and q have the same transcript length and so any differences to their translation rate will be due to the difference in the number of transcribed genes belonging to each fraction. The ratio of approximately 4:950 from [1] implies that q-transcripts are 238 times more abundant than e-transcripts. Given that a typical *E. coli* cell contains approximately 1,380 mRNA molecules in exponential growth [9], the ratio from [6] implies there are $\frac{1380}{954} \times 4 \approx 6$ enzymatic transcripts at steady state. Intuitively, this is too small, therefore we adjust this ratio to 20:850, which gives $\frac{1380 \times 20}{870} \approx 32$ enzymatic transcripts, a more reasonable value.

We additionally reduced the ribosome transcription rate, v_{TX_r} from 930 in [1] to 27 (unit: molecs min^{-1} cell^{-1}). This is because 68% of the cell's RNA polymerases work on ribosomal RNA synthesis [10] and

ribosomal mRNA is 30x longer than non-ribosomal mRNA in our framework. It follows that the ribosomal transcription rate is $\frac{68\%}{30} \approx 2.3\%$ of the total transcription rate of an *E. coli* cell. This suggests that *E. coli*'s total mRNA synthesis rate is approximately $\frac{27}{2.3\%} = 1174$ min^{-1} cell^{-1}, which is close to the sum of our constituent transcription rate parameters ($v_{\mathrm{TX}_r} + v_{\mathrm{TX}_e} + v_{\mathrm{TX}_q} = 897$ min^{-1} cell^{-1}). Our value is slightly lower as we wanted to account for the added effect of RNA polymerase traffic jams on ribosomal RNA genes [10].

S3.2 mRNA:protein mass ratio calculations

During our simulations, we calculate the mRNA:protein mass ratio for comparison with bacterial growth laws. We take the average molecular weight of a nucleotide to be 330 g mol^{-1} and of an amino acid as 118.9 g mol^{-1} [11]. The total mass of mRNA transcripts is then calculated as the sum of the mass of each transcript *type* multiplied by its corresponding abundance in the cell. The total protein mass was obtained similarly, giving the quantities needed for the mRNA:protein mass ratio.

S4 Supplementary results

S4.1 Proteome fraction changes in response to nutrient quality

In Figure 4 from the main text, we used our model to reproduce a number of 'bacterial growth laws' by showing how different variables changes in response to increasing nutrient quality, n. For the endogenous simulations (without inclusion of a synthetic gene construct), we showed that increasing n increased the growth rate to the point of saturation while monotonically increasing the mRNA:protein mass ratio. While not central to our main results, it may also be useful to observe what happens to the steady-state values of all the endogeneous proteome components: R, E and Q. We display the results below for the seven different values of n as used in Figure 4.

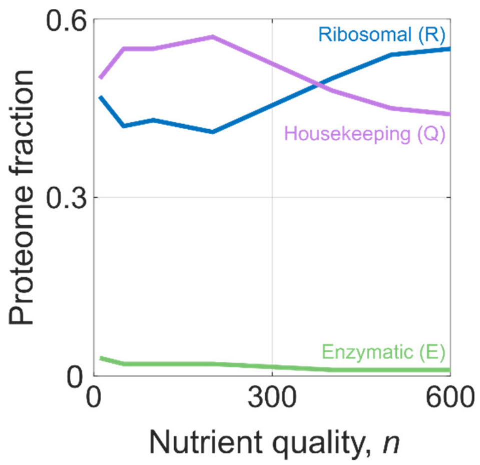

Figure S1. Steady-state endogenous proteome fractions plotted against nutrient quality.

From Figure S1, it can be seen that R and Q mirror each other's dynamics with R being the dominant species at high values of n , while E shows a slight relative decrease when n increases. This can be explained in terms of how the cell distributes its resources when it has access to a large energy supply: in higher nutrient quality conditions (i.e. at higher values of n), more energy is available to the cell per nutrient molecule absorbed and thus there is no need for the cell to absorb and metabolize as many nutrients as in lower nutrient quality conditions (smaller values of n). As a result, E decreases, and the cell has more resources to spend on other fractions. For increasing values of n, this causes Q to increase, due to its large maximal transcription rate. At even higher values of n, Q eventually gives space to R, due to the fact that mRNA species of Q are negatively autoregulated.

S4.2 Changing 'slow codon' parameters

In addition to the parameters mentioned in the main text, we changed three other key parameters to better understand the effect these had on the model's output:

i) *Slow codon position*. In addition to placing a slow codon towards the end of the mRNA transcript, we conducted simulations with a slow codon near the beginning of the transcript (footprint position 5 R_f). In this instance, translation is still slowed down, however only very short queues can form, leading to a lower amount of sequestered ribosomes.

ii) *Slow codon efficiency*. In addition to a slow codon that has a relative efficiency of 0.5%, we ran simulations with a slow codon with a relative efficiency of 3%. This was used to assess how changing the efficiency of a codon affects queue formation.

iii) *mRNA length*. Our standard simulations use mRNAs with a length of 30 R_f. In reality, mRNA length can vary significantly. We also ran simulations with mRNAs of length 60 R_f to test whether this has an impact on queue formation. In this instance, the slow codons were located at footprint positions 5 R_f or 56 R_f.

As the impact of these parameters was not the focus of our study, we provide just a basic snapshot of their effects, in the form of the relationship between H_{rate} and G_{rate} at steady state. In general, the more efficient the translation process is, the higher the values of G_{rate} are for equivalent values of H_{rate}. In addition, the linearity of each trend suggests how predictable the dynamics of translation and protein production are, with more linear trends conveying higher predictability. In this light, linear regression fits are provided for each case studied. Slower codons towards the end of the mRNA typically give rise to very inefficient constructs, depending on $prom_H$ and RBS_H.

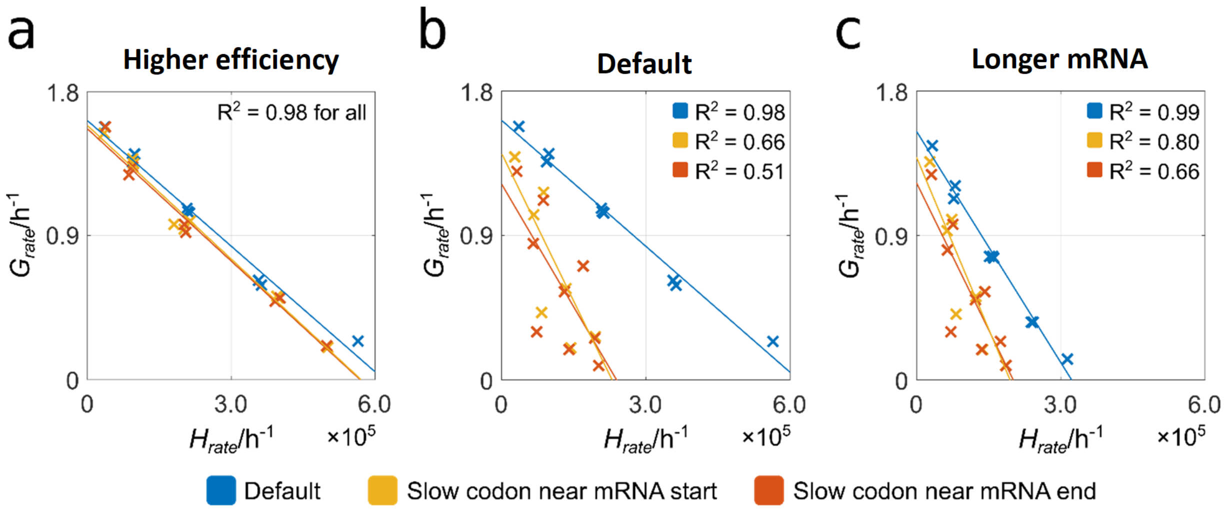

Figure S2. Comparing the impact of different parameters via their effect on the relationship between H_{rate} and G_{rate}. In each subfigure, a different parameter has been varied, and we include plots for each location of slow codon on the mRNA transcript: blue = no slow codon, yellow = slow codon near the start (at position 5 R_f), orange = slow codon near the end (at positions 26 R_f for shorter mRNA and 56 R_f for longer mRNA). R^2 values from a linear regression are given. **(a)** Slow codon efficiency = 3%, mRNA length = 30 R_f. **(b)** Slow codon efficiency = 0.5%, mRNA length = 30 R_f. **(c)** Slow codon efficiency = 0.5%, mRNA length = 60 R_f.

S4.3 Absolute values of heterologous protein yield

In Section 3.2.2, we presented an analysis of hypothetical growth scenarios and calculated how much heterologous protein would be produced over time. When presenting this data in Figure 6, we used a normalised form of protein yield ($H(t)_{\mathrm{norm}}$) at each time point in order to make comparisons between different construct designs easier to interpret. The absolute values were not included in the core manuscript, however it may still be useful for readers to see these. In Figure S3, we therefore present heat maps of *absolute* protein yield values for both growth scenarios (uncapped exponential growth, turbidostat growth) and for both of the main codon compositions used (no slow codons, one slow codon towards the end of a transcript). The interpretation of this figure is identical to that in Figure 6b, except we now:

- show the full suite of heat maps for both growth scenarios separately. This is because the absolute protein yield increases over time even when protein production dynamics are time-invariant, such as in a turbidostat operating at steady-state capacity
- use a separate scale bar for each heat map. This is in order to preserve visual comparisons between construct designs at a specific time interval

For subfigure a, the shading of heat maps over the time intervals is identical to those in Figure 6b. In subigure b, the shading of each heat map remains consistent over the time points because the protein production dynamics of a turbidostat operating at steady-state capacity remain constant over time.

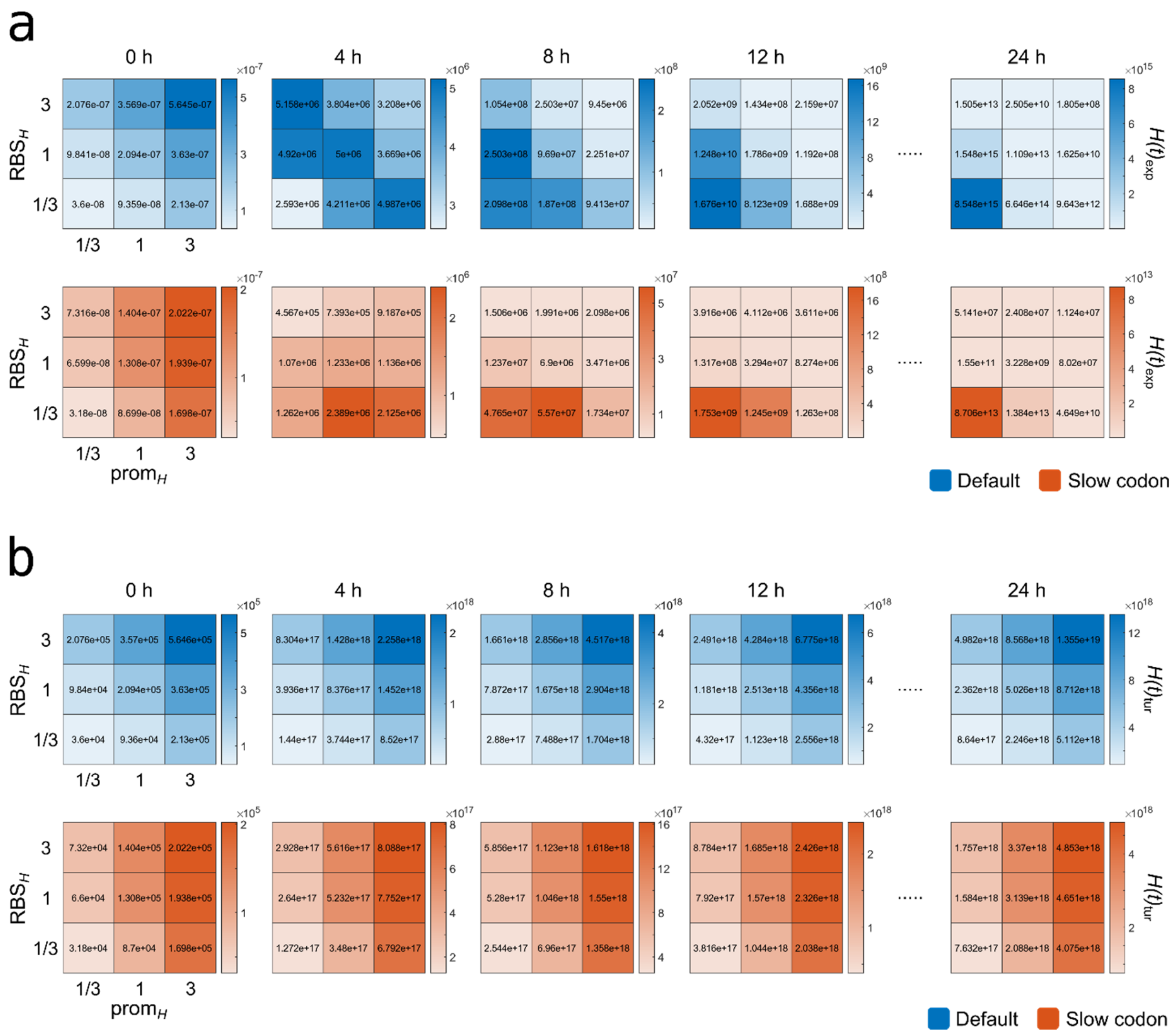

Figure S3. Absolute values of heterologous protein yield for a variety of construct design and growth conditions. Values from different promoter and RBS strengths are displayed using 3x3 matrices. Values from different codon compositions are shown via differently coloured rows (blue: no slow codon; orange: one slow codon towards the transcript's end). Values from different growth scenarios are split into the two subfigures (**a**: uncapped exponential; **b**: turbidostat operating at steady-state capacity). For a more thorough interpretation of the figure's design, see the caption for Figure 6 in the main manuscript.

S4.4 Simulation convergence, ribosome densities and proteome mass fractions

For each combinaiton of promoter strength and RBS strength, we show three set of additional data: (i) the convergence of the simulation, (ii) the ribosome density on $mRNA_H$ transcripts, and (iii) the steady-state proteome mass fractions. For each case, we show a set of data for scenarios when the slow codon (codon efficiency = 0.5%) is positioned near the beginning of an $mRNA_H$ transcript of length 30 R_f (at position 5 R_f), and near the end of that trancript (at position 26 R_f). This results in two sets of nine graphs (for each promoter-RBS combination tested) in each case. Within each set, the subplots are arranged in a 3x3 grid to match the promoter-RBS values from Figures 5a,b and Figurse 6a,b.

Simulation convergence

As stated in Section 2.1, a simulation stops when all variables have reached convergence. This is defined as when a variable's quantity does not deviate by more than 1% from its moving mean, where the moving mean is calculated from the last 10% of the simulation time. Below, we show how this is performed for the heterologous protein species (H).

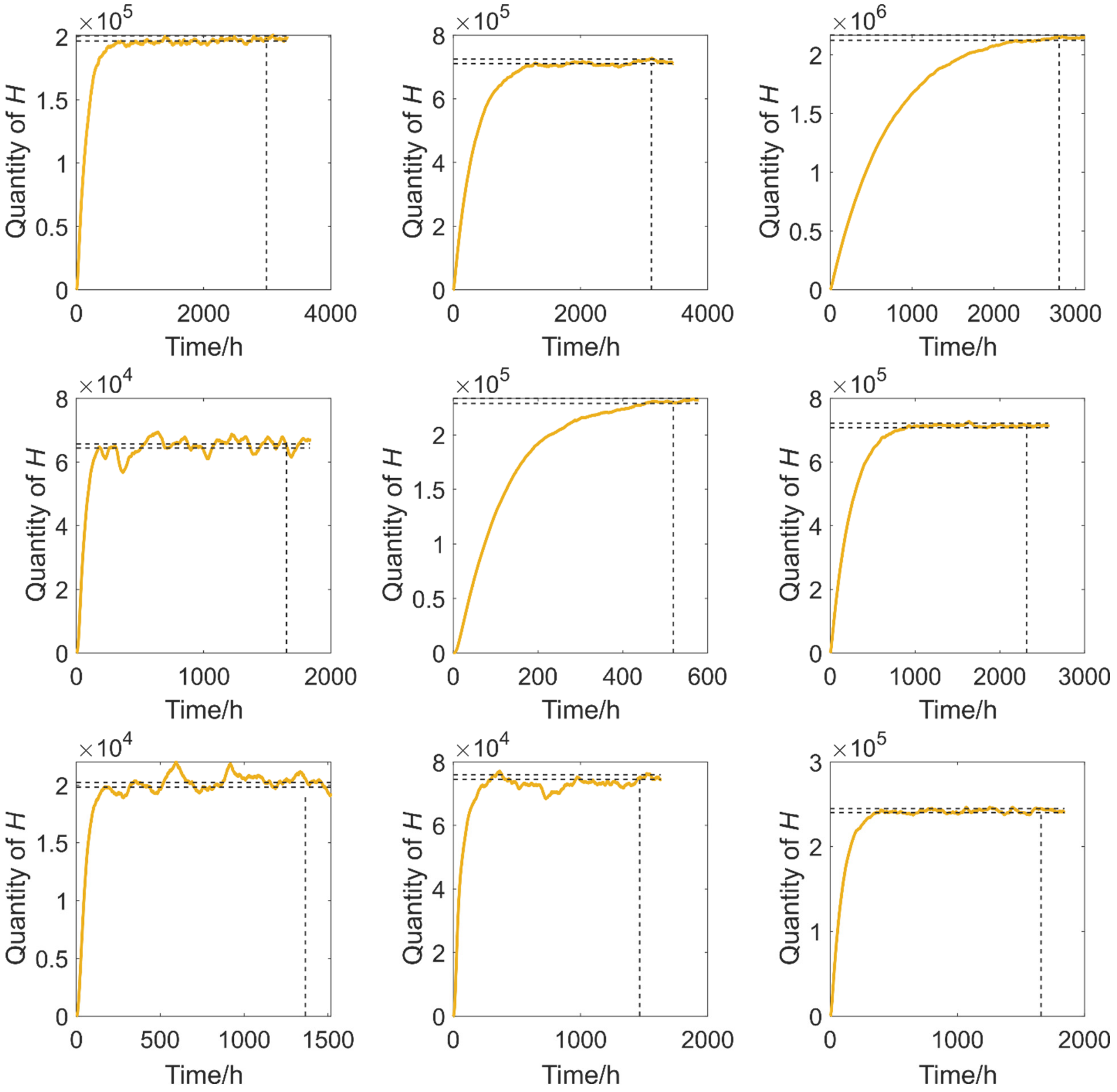

Figure S4. Convergence of the heterologous protein variable, H, for simulations with a slow codon (codon efficiency = 0.5%) at position 5 R_f out of 30 R_f. Lines represent the quantity of H during the course of a simulation with particular values for promoter strength and RBS strength, as defined at the start of Section 4.4. Horizontal dashed lined denote 1% deviations from the mean value of H during the last 10% of simulation time. The vertical dashed line refers to the time at 90% of the total simulation time.

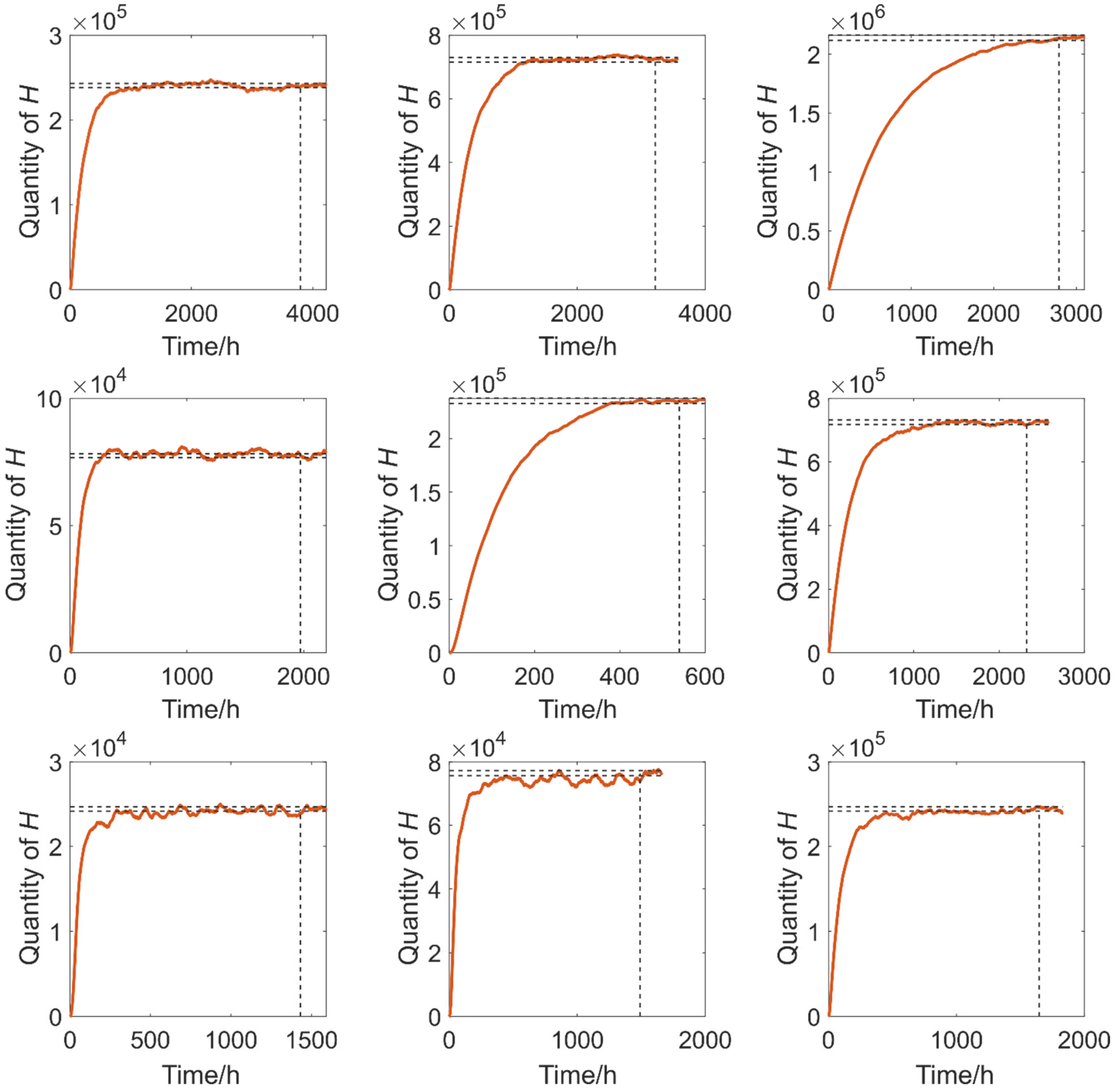

Figure S5. Convergence of the heterologous protein variable, H, for simulations with a slow codon (codon efficiency = 0.5%) at position 26 R_f out of 30 R_f. The definition of the dashed lines is identical to that of Figure S3.

Ribosome density plots

As explored in Section 3.2.1 and in Figure 5d, we can plot the proportion of ribosomes on $mRNA_H$ transcripts that are on each footprint position.

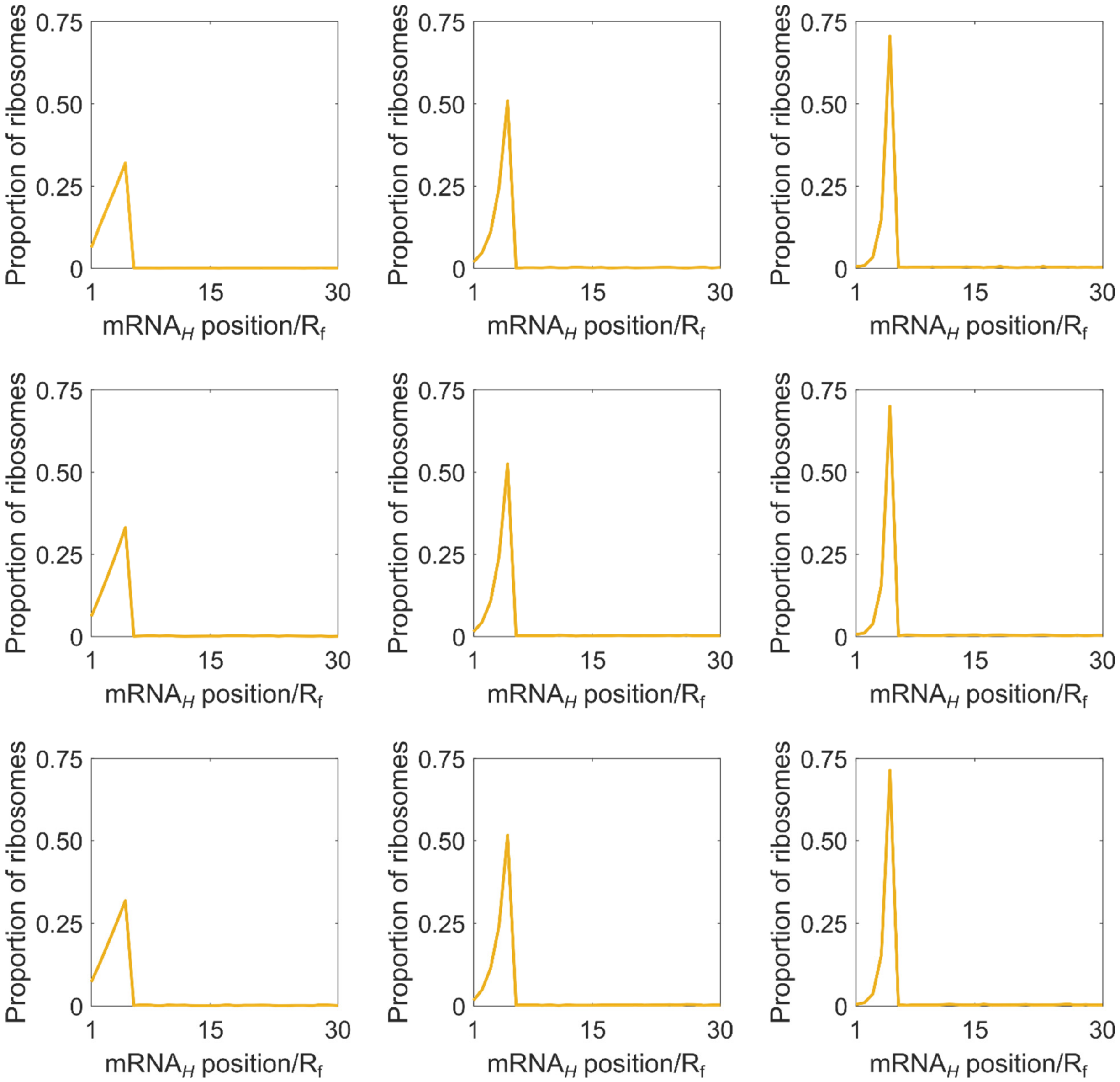

Figure S6. Ribosome density plots for simulations with a slow codon (codon efficiency = 0.5%) at position 5 R_f out of 30 R_f.

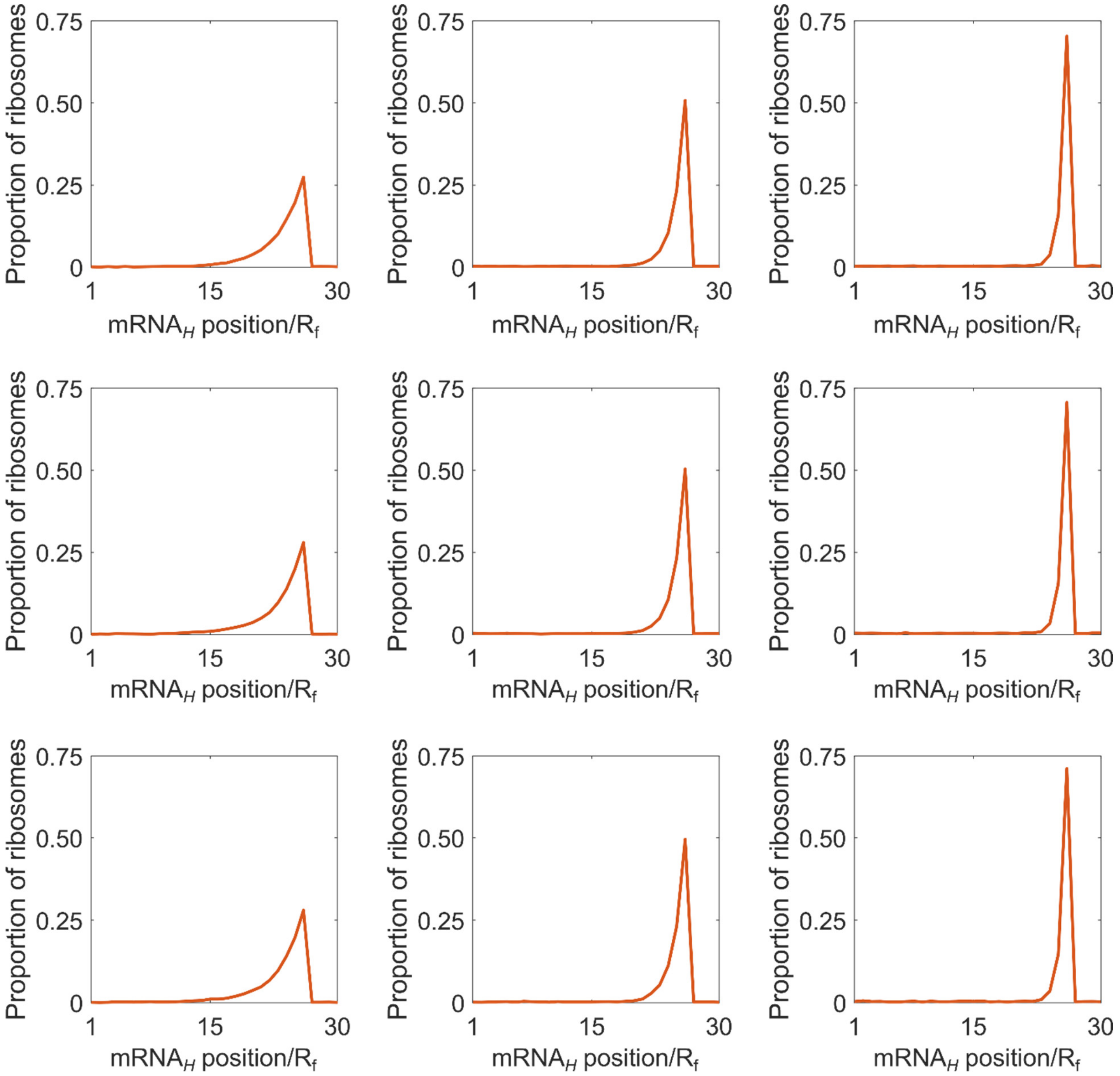

Figure S7. Ribosome density plots for simulations with a slow codon (codon efficiency = 0.5%) at position 26 R_f out of 30 R_f.

Proteome mass fractions

Finally, we plot the steady-state mass fractions of each proteome class, and represent these as pie charts.

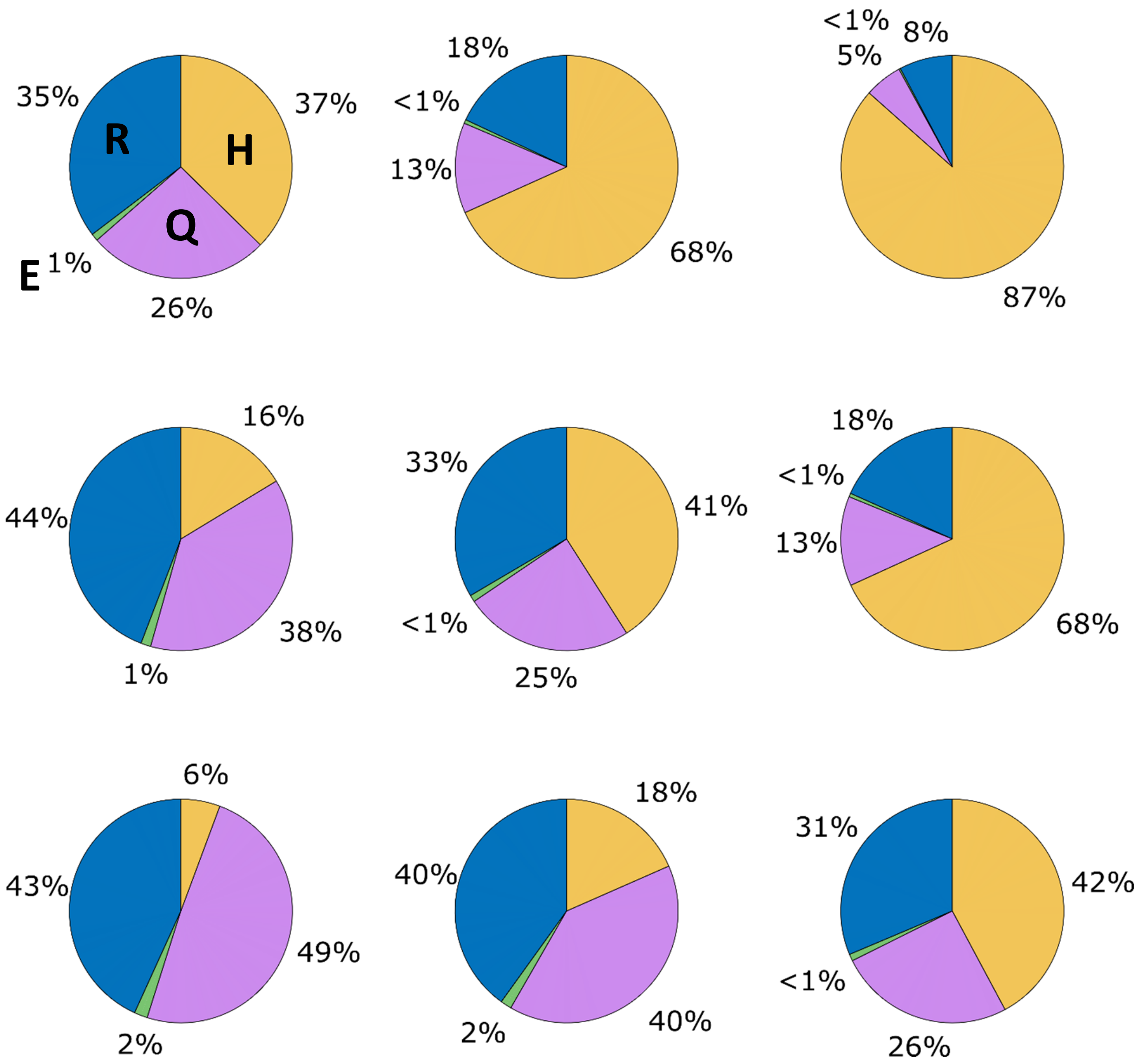

Figure S8. Steady-state proteome mass fractions for each protein class for simulations with a slow codon (codon efficiency = 0.5%) at position 5 R_f out of 30 R_f. Blue = R, green = E, purple = Q, yellow = H.

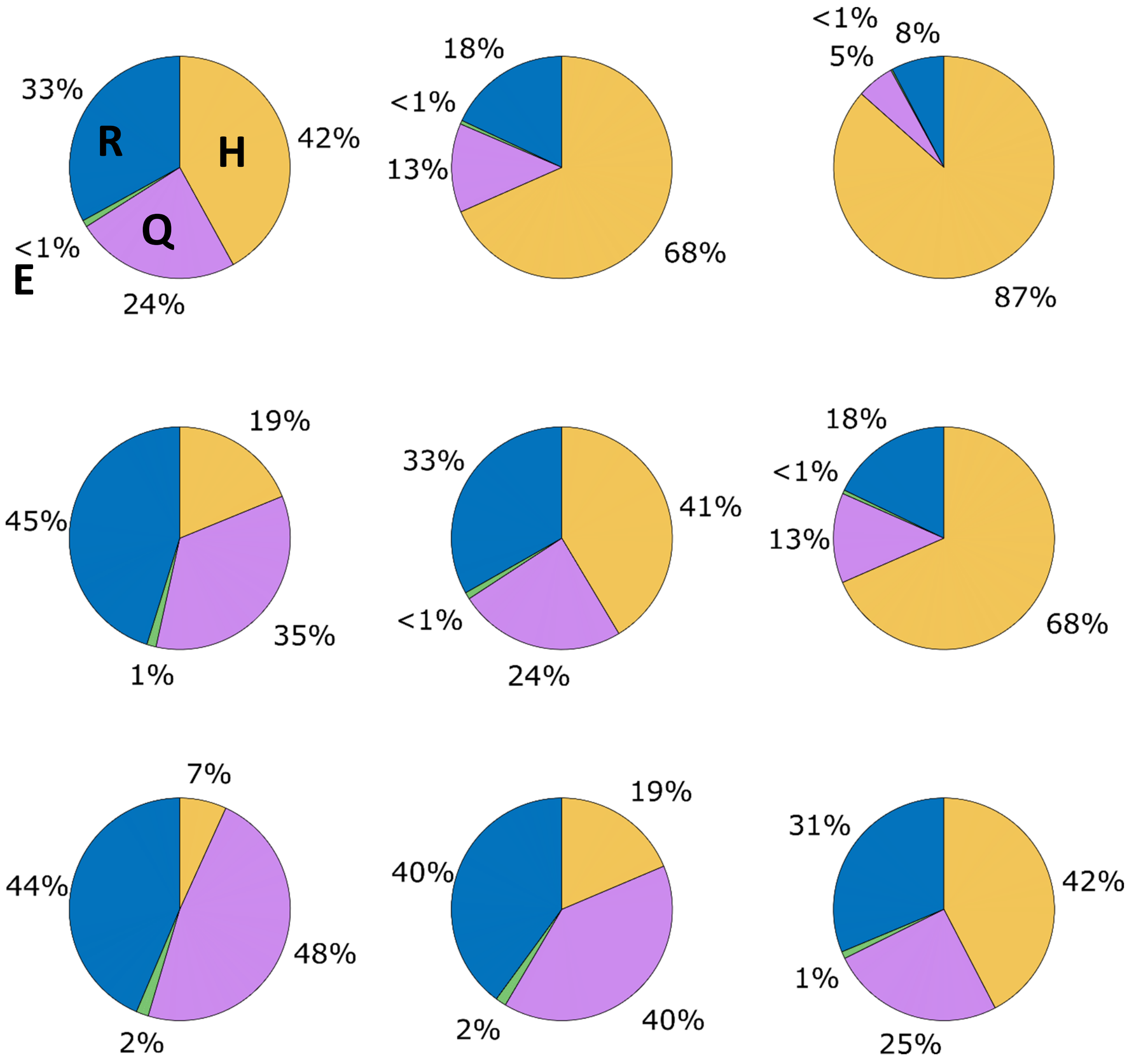

Figure S9. Steady-state proteome mass fractions for each protein class for simulations with a slow codon (codon efficiency = 0.5%) at position 26 R_f out of 30 R_f. Blue = R, green = E, purple = Q, yellow = H.

References

1. Weiße AY, Oyarzún DA, Danos V, Swain PS. Mechanistic links between cellular trade-offs, gene expression, and growth. Proceedings of the National Academy of Sciences. 2015 Mar 3;112(9):E1038-47.
2. Bremer HD, Dennis PP. Modulation of chemical composition and other parameters of the cell by growth rate. Escherichia coli and Salmonella: cellular and molecular biology. 1996 Jan;2(2):1553-69.
3. Keseler IM, Collado-Vides J, Santos-Zavaleta A, Peralta-Gil M, Gama-Castro S, Muñiz-Rascado L, Bonavides-Martinez C, Paley S, Krummenacker M, Altman T, Kaipa P. EcoCyc: a comprehensive database of Escherichia coli biology. Nucleic acids research. 2010 Nov 20;39(suppl_1):D583-90.
4. Brandt F, Etchells SA, Ortiz JO, Elcock AH, Hartl FU, Baumeister W. The native 3D organization of bacterial polysomes. Cell. 2009 Jan 23;136(2):261-71.
5. Algar RJ, Ellis T, Stan GB. Modelling essential interactions between synthetic genes and their chassis cell. In53rd IEEE Conference on Decision and Control 2014 Dec 15 (pp. 5437-5444). IEEE.
6. Taniguchi Y, Choi PJ, Li GW, Chen H, Babu M, Hearn J, Emili A, Xie XS. Quantifying E. coli proteome and transcriptome with single-molecule sensitivity in single cells. science. 2010 Jul 30;329(5991):533-8.
7. Strange RE. Bacterial "glycogen" and survival. Nature. 1968 Nov;220(5167):606-7.
8. Sanchez-Vazquez P, Dewey CN, Kitten N, Ross W, Gourse RL. Genome-wide effects on Escherichia coli transcription from ppGpp binding to its two sites on RNA polymerase. Proceedings of the National Academy of Sciences. 2019 Apr 23;116(17):8310-9.
9. Neidhardt FC. Escherichia Coli and Salmonella: Cellular and Molecular Biology. 1996;Vol 1. ASM Press.
10. Klumpp S, Hwa T. Traffic patrol in the transcription of ribosomal RNA. RNA biology. 2009 Sep 1;6(4):392-4.
11. Milo, R., Jorgensen, P., Moran, U., Weber, G. and Springer, M., 2010. BioNumbers—the database of key numbers in molecular and cell biology. Nucleic acids research, 38(suppl_1), pp.D750-D753.
